# Effects of post-traumatic growth on the dorsolateral prefrontal cortex after a disaster

**DOI:** 10.1038/srep34364

**Published:** 2016-09-27

**Authors:** Seishu Nakagawa, Motoaki Sugiura, Atsushi Sekiguchi, Yuka Kotozaki, Carlos Makoto Miyauchi, Sugiko Hanawa, Tsuyoshi Araki, Hikaru Takeuchi, Atsushi Sakuma, Yasuyuki Taki, Ryuta Kawashima

**Affiliations:** 1 Institute of Development, Aging and Cancer (IDAC), Tohoku University, Sendai, Japan; 2 Division of Psychiatry, Tohoku Medical and Pharmaceutical University, Sendai, Japan; 3 International Research Institute of Disaster Science, Tohoku University, Sendai, Japan; 4 Division of Medical Neuroimage Analysis, Department of Community Medical Supports, Tohoku Medical Megabank Organization, Tohoku University, Sendai, Japan; 5 Department of Adult Mental Health, National Institute of Mental Health, National Center of Neurology and Psychiatry, Kodaira, Tokyo, Japan; 6 Graduate School of Arts and Sciences, The University of Tokyo, Tokyo, Japan; 7 Advantage Risk Management Co., Ltd., Tokyo, Japan; 8 Department of Psychiatry, Tohoku University Graduate School of Medicine, Sendai, Japan

## Abstract

The relating to others factor of post-traumatic growth (PTG), which involves mutual help and a strong sense of connection with humanity, is important for young people who are coping with stress. The prefrontal cortex (PFC), especially the dorsolateral PFC (DLPFC), may play an important role in post-traumatic stress disorder (PTSD) with regard to coping and resilience. We hypothesized that the neural correlates of PTG may be responsible for resilience to the correlates of PTSD. Our study tested this hypothesis by examining whether measures of PTG, particularly the measures of relating to others after a disaster, were associated with increased regional grey matter volume (rGMV) in the PFC by assessing individuals who had experienced the East Japan Great Earthquake. We calculated the delta-rGMV by subtracting the rGMV obtained 3 months before the disaster from the rGMV obtained after this disaster using voxel-based morphometry. The magnetic resonance imaging data obtained from 26 subjects (M/F: 21/5; age: 21.2 ± 1.6 yrs.) showed that the total scores on a PTG inventory and the subscore for relating to others at the post-assessment were positively and significantly associated with the delta-rGMV in the right DLPFC. The DLPFC seems to be the main neural correlate of PTG.

Post-traumatic growth (PTG) is characterised by subjective, positive psychological changes resulting from major life crises or traumatic events^1^. Increases in appreciation of life, personal resilience, the quality of intimate relationships, and spiritual wellbeing, as well as the resetting of life priorities and openness to new possibilities, are typical of these positive psychological changes^2^. In PTG, successful coping in the aftermath of a traumatic event occurs when an individual’s perceptions of the self, others, and the meaning of the event are positively reconstructed^2^.

In the present study, a particular focus was placed on the relating to others factor of the PTG Inventory (PTGI) because Japanese individuals live in a collective culture and do not tend to report personal strengths^3^. The relating to others factor is associated with significant changes in attitudes towards subjective relationships, such as increased compassion, intimacy, and closeness^2^. Relating to others is crucial for individuals who are coping with stress because relationships serve as a buffer against the negative effects of stressful experiences^4^. When mutual support is available to meet the needs of individuals coping with stress, they respond in more active, flexible, and positive ways^4^. Accordingly, relating to others may mitigate the impact of negative experiences. For example, relating to others has a significant negative correlation with the degree of chronic fatigue^5^. Increased ability to relate to others promotes willingness to accept help when in need, as well as utilisation of social support that had previously not been exploited^2^. These positive psychological changes appear to be effective in preventing cumulative fatigue.

Interestingly, the type of traumatic event, and the age of the individual who experiences it, were found to modify the relationship between PTG and post-traumatic stress disorder (PTSD) symptoms significantly in a meta-analysis^6^. Based on an apparent inverted “U” curvilinear relationship between stress and PTG, subjective or objective stress might need to reach a certain level of severity to result in PTG^7^. A stronger relationship between PTG and PTSD symptoms has been observed in younger versus older people^6^,^8^. Furthermore, a significant relationship between PTG and PTSD was also seen in survivors of natural disasters^6^,^8^,^9^, although the majority of cross-sectional studies found no significant relationship between these two phenomena^10^.

In this study, we focused on activation in the prefrontal cortex (PFC), especially the dorsolateral PFC (DLPFC), in terms of its potential relationship with PTG. The evidence for DLPFC involvement in recovery from PTSD, resilience, and coping is as follows: greater thickness of the DLPFC was observed in subjects who experienced trauma compared with controls, which in turn was associated with reductions in PTSD symptoms and better recovery^11^. In terms of resilience, asymptomatic traumatised control participants who all experienced the same trauma showed thicker DLPFCs than both participants with PTSD and control participants drawn from among the general population^12^. A functional imaging study showed that increased activation in regions of the PFC and dorsal anterior cingulate cortex (ACC) was associated with resilience in response to stress^13^. With regard to stress coping mechanisms, the PFC plays an important role in translating stressful experiences into adaptive behaviour by integrating cognitive and affective behaviours, as well as facilitating neuroendocrine and autonomic flexibility in response to stress^14^.

Furthermore, the DLPFC may be associated with the ability to relate to others; empathy and emotional/social intelligence also appear to be involved in this ability^2^. Indeed, an increase in resting-state functional connectivity between the anterior insula and the middle part of the right DLPFC was observed in subjects with higher levels of emotionality and social intelligence^15^.

According to the evidence presented by the above neuroimaging study, we hypothesised that the PFC (particularly the DLPFC), together with functionally and structurally related brain regions, may play an important mechanistic role in PTG, especially in terms of relating to others.

The Great East Japan Earthquake was a massive 9.0+-magnitude (Richter scale) earthquake that occurred in the Pacific Ocean near northeastern Japan on March 11, 2011 and caused serious damage to almost all of the Tohoku area. Thus, undergraduate and postgraduate students at Tohoku University and young residents who experienced the earthquake were recruited to the present study.

Although many studies have investigated the neural correlates of post-traumatic stress (PTS), including those of PTSD^16^,^17^, to date, no studies have attempted to determine the neural correlates of PTG. Such studies may be important because the decreased activation observed in cortical regions associated with PTSD may be ameliorated based on the degree of PTG^11^. It is possible that the attenuation of cortical loss due to PTG may be a mechanism underlying the resilience to the effects of PTSD, which is consistent with previous findings showing that reductions in cortical loss accompany improvements in PTSD symptoms^11^,^18^. Regional grey matter volume (rGMV) is defined as a certain regional volume of a high density of heavily interconnected neurons, which is the basis of sensation, thought and action^19^. Accordingly, an increase in rGMV indicates an increase a certain regions of interconnected neurons, which lead to human activity. Thus, in the present study, it was hypothesized that the total score on the PTGI and the subscore for relating to others may be associated with increased rGMV and resilience to the effects of PTSD after a disaster. This hypothesis was tested by examining whether the total score on the PTGI and the subscore for relating to others obtained at 3 months after the earthquake disaster (post) were associated with an increase in rGMV in the PFC (particularly the DLPFC) and regions that are structurally and functionally related to the PFC compared to the levels observed before the disaster (pre; i.e., delta-rGMV) in residents of Miyagi Prefecture.

Moreover, a strong relationship between PTG and PTSD symptoms was previously observed in young people exposed to natural disasters^6^. Thus, the present study also investigated the relationship between PTG and PTSD symptoms.

## Results

### Psychological data

**Figure 1:**
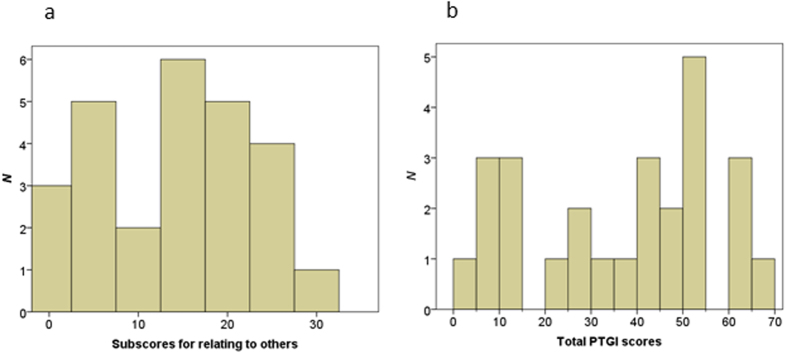
Distribution of the total PTGI scale scores.

The mean (±SD) total score on the Raven’s Advanced Progressive Matrix (RAPM) was 30 ± 4.4. Table 1 shows the mean (±SD) scores on the four subscales of the PTGI, the Clinician-administered PTSD Scale (CAPS), the Center for Epidemiologic Studies Depression Scale (CES-D), and the Trait Anxiety (T-A) subscale of the State–Trait Anxiety Inventory (STAI). The scores on the four subscales of the PTGI were significantly and positively correlated with each other (*P* < 0.01, Bonferroni-corrected). Figure 1 shows the distribution of total PTGI scale scores. The internal reliability of the four subscales of the PTGI was high (Cronbach’s alpha, 0.80–0.88).

### Imaging data

**Figure 2:**
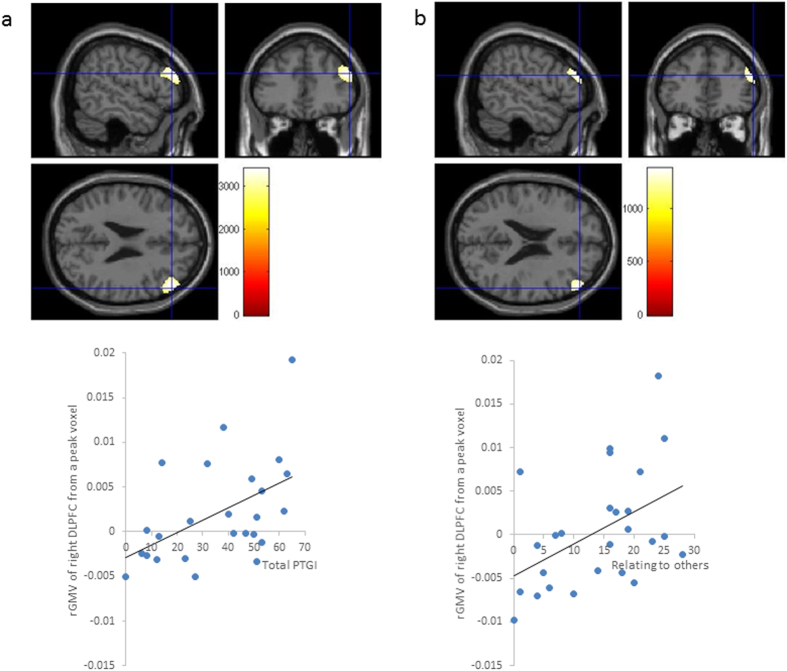
(**a**) Regions that were correlated with the delta-rGMV values from the significant peak voxel. After controlling for sex, age, and total intracranial volume (TIV), the subscore for relating to others was positively associated with the delta- rGMV in the right dorsolateral prefrontal cortex (DLPFC) (x = 54, y = 38, z = 25; Threshold-free cluster enhancement [TFCE] = 3405.80, *P* = 0.027, k = 917), with a family-wise error (FWE) correction. A scatterplot of the relationship between the scores for relating to others and the delta-rGMV value from the significant peak voxel is shown. (**b**) Regions that were correlated with the delta-rGMV values from the significant peak voxel and total score on the PTGI. After controlling for sex, age, and TIV, the total score on the post-traumatic growth inventory (PTGI) was positively associated with the delta-rGMV in the right DLPFC (x = 54, y = 42, z = 22; Threshold-free cluster enhancement [TFCE] = 1376.47, *P* = 0.031, k = 504), with a family-wise error (FWE) correction through small volume correction (SVC) using a mask with regions that are functionally and structurally related to the DLPFC. A scatterplot of the relationship between the total scores on the PTGI and the delta-rGMV value from the significant peak voxel is shown.

After controlling for sex; age; total intracranial volumes (TIV; total GMV+ total white matter volume [WMV]+ total cerebrospinal fluid volume); total scores on the RAPM, CAPS, and CES-D; score on the T-A subscale of the STAI at post; and the interval between the pre- and post-magnetic resonance imaging (MRI) data acquisitions, the whole-brain analysis revealed delta-rGMV in the right DLPFC was significantly correlated to the subscore for relating to others (x = 54, y = 38, z = 25; threshold-free cluster enhancement [TFCE] = 3,405.80, *P* = 0.027, k = 917, with family-wise error [FWE] correction) (Fig. 2a).

When the analyses were limited to brain regions that are structurally or functionally related to the DLPFC and controlled for the abovementioned covariates, the regression analysis showed that the total score on the PTGI was positively associated with the delta-rGMV in the right DLPFC (x = 54, y = 42, z = 22; TFCE = 1,376.47, *P* = 0.031, k = 504, with FWE corrected through small-volume correction [SVC]) using the mask-related regions that were functionally and structurally related to the DLPFC (Fig. 2b).

The results of the regression analyses showed that the total score and relating to others subscore on the PTGI were significantly and positively correlated with the peak of the statistically significant delta-rGMV (right DLPFC). The peak of the statistically significant delta-rGMV was defined as the highest significant change in grey matter volume in a voxel from the pre to post measurements. In contrast, the total score on the CAPS showed a significant negative correlation with the value of the statistically significant delta-rGMV that was identified using the peak voxel (Table 2). Multicollinearity seemed to be ruled out because all of the variance inflation factors (VIFs) and the beta values for the total PTGI were less than 4 (Table 2). The overall fitness in the regression analyses was good enough (*R*^2^ > 0.5).

After controlling for the abovementioned covariates using the post rGMV measurements, neither the whole brain nor regions of interests (ROIs) (using SVC) multiple regression analyses identified significant relationships for the total score on the PTGI or the subscore for relating to others.

## Discussion

The present study showed that the total score and the relating to others subscore on the PTGI were associated with an increased rGMV (i.e., delta-rGMV) in the right DLPFC after the subjects experienced a disaster compared to the values obtained before the disaster. This finding is in accord with the hypothesis that the neural correlates of PTG may represent a mechanism that is associated with resilience to the correlates of PTSD. More specifically, the DLPFC plays an important role in the recovery from PTS^11^, resilience^13^, coping^20^, and the response to stress. Interestingly, in terms of compensation, which is a means of coping with stress, the DLPFC is more highly activated after total sleep deprivation than after normal sleep^21^. A model of PTG suggests that the struggle to recover in the aftermath of a disaster often yields positive growth^2^, and it is possible that this struggle may be reflected in the association between PTG, particularly relating to others, and increased rGMV in the DLPFC.

It is important to determine why the DLPFC, but not other candidate regions, was identified as a neural correlate of PTG. Previously, a quantitative meta-analysis observed that there were consistent GM reductions in the ACC, ventromedial PFC (VMPFC), and left middle temporal gyrus (MTG) regions of patients with PTSD compared to individuals who were exposed to trauma but did not develop PTSD^22^. The subjects in the present study were healthy subjects without PTSD, and thus, this selection bias might be the reason why the ACC, VMPFC, and left MTG were not significantly related to PTG. Moreover, the DLPFC has an important role in conflict-induced behavioural adjustments^23^, whereas the ACC does not seem to play an indispensable role in behavioural adjustments because it monitors conflict by receiving relevant or irrelevant as stimuli at the sensory level^23^. From a developmental point of view, the DLPFC is the final region to mature within the PFC, towards the end of adolescence^24^. There is a deactivation of the DLPFC in adolescents under conditions of high stress versus low stress, whereas adults exhibit a greater activation of the DLPFC during periods of high stress^20^. Accordingly, the rGM in the DLPFC regions of the young subjects in the present study may have been more sensitive to PTG than the rGM of older subjects. From the perspective of therapeutic strategies, the DLPFC provides the optimal prerequisite for successful non-invasive brain stimulation techniques, such as repetitive transcranial magnetic stimulation (rTMS), for patients with psychiatric disorders, including depression^25^. Interestingly, rTMS to the DLPFC induces increased prosocial behaviour in all emotional situations^26^. Additionally, our research group found that a greater degree of PTSD symptoms following the earthquake was associated with a lower GMV in the ACC before the earthquake^27^. Thus, although it may have acted as a predisposing factor, the GMV in the ACC was not significantly related to the reduction in PTSD symptoms between the pre- and post-assessments in the present study^27^. In this manner, the DLPFC seems to be more involved in adaptive growth than the ACC, which may be related to the effects of empathic responses to this extraordinary disaster.

It is also important to consider that the subjects with lower scores for relating to others showed reduced rGMV in the DLPFC. Acute and chronic stress influence the brain by altering dendritic spine density and length, as well as dendritic branching in several brain regions, including the PFC^28^. Generalized increases in calcium-cyclic adenosine monophosphate (AMP) signalling during fatigue or stress disengage the DLPFC recurrent circuits and impair cognition^29^. Accordingly, the reduced rGMV in the DLPFC that was observed in the present study may have been associated with lower scores for relating to others and may represent the neurological correlate of having little sense of a connection with humanity.

There were no significant correlations among the four subscales of the PTGI and the CAPS in the present study (Table 1). This finding is in accordance with the findings of a previous study that analysed the 2-year follow-up survey data of 1,057 US military veterans and found that the effects of PTG in patients with PTSD are relatively small and that causality cannot be inferred^30^. On the other hand, the total score on the CAPS had a significant negative relationship with the value of the delta-rGMV in the right DLPFC (Table 2). In other words, PTG and negative post-trauma outcomes could co-occur in our subjects, based on the multiple regression analyses. This outcome is in accordance with a meta-analysis showing a strong relationship between PTG and PTSD symptoms^6^. Moreover, a study that evaluated the 1-year follow-up data of 165 adolescent and young adult cancer patients showed a curvilinear relationships between PTS symptoms and two PTG factors (new possibilities and personal strengths)^31^. As mentioned in the Introduction, this partial discrepancy might be due to the collectivist values of the Japanese culture^3^,^5^, and differences in age and type of traumatic event^6^.

Several limitations of this study should be noted. As participants were not directly or severely damaged by the disaster, the present results may not be generalizable to victims who are directly exposed to life-threatening experiences. Our research group has not researched the real life events of the subjects surrounding the disaster or the extent to which the disaster truly affected each subject. However, as discussed in a previous report^5^, the total PTGI and subscale scores seem to be consistent with previous studies of PTG, and all subjects who lived around Sendai were strongly affected by the Great East Japan Earthquake^5^. In fact, the students at Tohoku University who lived in or around Sendai city (the same regions as our subjects) exhibited psychological stress by an increase in salivary cortisol levels at 3 months after the earthquake compared with the levels before the earthquake^32^. Accordingly, our subjects seemed to experience something that truly elicited PTG. The possibility of selection bias should also be considered. Because the subjects were undergraduate and postgraduate students at Tohoku University, the present results may only be generalizable to well-educated members of the younger generation. Additionally, control subjects were not included because almost all individuals residing near the University were affected by the disaster to at least some extent. Moreover, the exclusion of subjects with mental disorders may have resulted in a failure to recognize other important neural correlates, such as other regions that are structurally and functionally related to the DLPFC. As we explained in our previous study^5^, although 3 months might be too brief to observe changes in new possibilities and spiritual changes, most changes in PTG occurred between 2 weeks and 2 months^33^. Finally, the small number of subjects in the present study reduced the chances of detecting an effect on the whole brain.

In conclusion, this is the first study to show that changes in the rGMV may reflect resilience and coping in response to stress, including reliance on compensation and relating to others, a factor of PTG. Importantly, the results showing increased rGMV in the right DLPFC provide new evidence of PTG in response to disasters. Further longitudinal investigations using larger and more diverse samples are needed to examine whether consistent brain alterations are related to PTG.

## Methods

### Subjects

The present study included 26 subjects (M/F: 21/5; age: 21.2 ± 1.6 yrs.) who were undergraduate and postgraduate students at Tohoku University. All subjects had participated in previous MRI experiments in our laboratory (i.e., pre; mean days before the disaster: 116 ± 34), were recruited again 3 months after the disaster (i.e., post; mean days after the disaster: 104 ± 9), and underwent subsequent structural MRI scanning. Because all candidates lived in the vicinity of the city of Sendai, which was strongly affected by the earthquake, we could not recruit control subjects. All subjects were right-handed, as assessed by the Edinburgh Handedness Inventory^34^, and all were screened for neuropsychiatric disorders using the Mini-International Neuropsychiatric Interview (M.I.N.I.)^35^,^36^ after the earthquake. The results of the M.I.N.I. confirmed that no subjects were exposed to life-threatening trauma due to the earthquake and tsunami and that no subject had a history of psychiatric illness. All participants were also interviewed by trained psychologists using the Japanese version of the structured interview from the Clinician-Administered post-traumatic stress disorder (PTSD) Scale (CAPS)^37^,^38^. Hence, no subject met the criteria for PTSD according to the M.I.N.I. or CAPS (7.7 ± 11.1; highest score: 31). Because no patients were assessed in this study, it was not considered a clinical investigation. Written informed consent was obtained from each subject and all methods in this study were performed in accordance with the Declaration of Helsinki (1991). This study was approved by the Ethics Committee of Tohoku University.

### Assessments and imaging acquisition

All psychological measurements were performed after the earthquake (i.e., post).

#### Post-traumatic growth (PTG) assessment

The Post-traumatic Growth Inventory (PTGI) was administered only after the earthquake because the imaging data were obtained from subjects who had been recruited for a separate study that was conducted prior to the disaster^39^. The PTGI was administered with an anchoring question that specifically referred to the earthquake. We informed all subjects that the title of this social brain study was ‘effects of this great widespread destruction disaster on cognition and behaviour, including changes in brain structure’ using a document and instructed them to answer the questions about this disaster. The PTGI appears to have utility for determining how successful individuals are in coping with the aftermath of a traumatic and stressful event^2^. The original PTGI is a 21-item scale that evaluates the success of a subject in coping with the aftermath of a trauma by measuring the degree of positive change in terms of reconstructing or strengthening perceptions of self, others, and the meanings of events^2^. The present study employed the Japanese version of the PTGI^40^, which is comprised four factors. In this study, a particular focus was placed on the social factor relating to others, which includes the following items: “Knowing that I can count on people in times of trouble”; “A sense of closeness with others”; “A willingness to express my emotions”; “Having compassion for others”; “Putting effort into my relationships”; “I learned a great deal about how wonderful people are”; and “I accept that I need others.” In addition to the relating-to-others factor, the other factors of the PTGI (new possibilities, personal strength, and spiritual change and appreciation of life) were also assessed^40^. All items were rated on a 6-point Likert scale that ranged from 0 (*not at all*) to 5 (*to a very great degree*).

#### Depression assessment

The Center for Epidemiologic Studies Depression Scale (CES-D) was developed to assess the epidemiology of depressive symptoms, including demonstrable sensitivity to significant life events, in the general population^41^,^42^. In the present study, the Japanese version of the CES-D, which contains 20 items that are rated on a 4-point scale ranging from 0 (*rarely or never*) to 3 (*most or all of the time*), was utilized^41^.

#### Anxiety assessment

In the present study, anxiety was assessed using the Trait Anxiety (T-A) subscale of the Japanese version of the State–Trait Anxiety Inventory (STAI)^43^,^44^,^45^. The T-A subscale evaluates relatively stable aspects of anxiety proneness, including general states of calmness, confidence, and security, using 20 trait anxiety items that are each rated on a 4-point scale: 1 (*almost never*), 2 (*sometimes*), 3 (*often*), and 4 (*almost always*)^25^.

#### Psychometric measures of general intelligence

Raven’s Advanced Progressive Matrix (RAPM), which is considered one of the best measures of general intelligence^46^, was administered to all subjects in the present study, and the results were adjusted for the effects of general intelligence on brain structures^47^,^48^,^49^. This measure was also used to exclude the possibility that any significant correlation between the regional grey matter volume (rGMV) and relating to others was caused by an association between general intelligence and relating to others or rGMV.

### Image acquisition

All MRI data were acquired with a 3-T Philips Intera Achieva scanner(Philips Medical Systems, Best, Netherlands) using a Magnetization Prepared Rapid Acquisition Gradient Echo (MPRAGE) sequence. High-resolution T1-weighted structural images (240 × 240 matrix, TR = 6.5 ms, TE = 3 ms, FOV = 24 × 24 cm^2^, 162 slices, 1.0-mm slice thickness) were collected.

### Analysis

#### Psychological data analysis

Pearson’s product correlations were used to examine the relationships among the scores on the subscales of the PTGI, total scores on the CAPS and CES-D, and score on the T-A subscale of the STAI (*P* < 0.05 [two-tailed] after Bonferroni’s correction). All behavioural data were analysed with the SPSS for Windows software package (ver. 22.0; IBM Corp., Armonk, NY, USA).

#### Neuroimaging data analysis

All analyses were conducted using MATLAB 7.6 (MathWorks, Natick, MA, USA).

Many imaging researchers use voxel-based morphometry (VBM) within Statistical Parametric Mapping (SPM) packages^50^. VBM was performed to identify the structural changes in the rGMV from pre- to post-assessment that may be attributed to survivors without clinical symptoms. First, the scans obtained during both pre- and post-assessments were coregistered to a single T1 image on SPM8.

Second, the T1-weighted structural images of each subject were segmented into grey matter (GM), white matter (WM), and cerebrospinal fluid using the standard unified segmentation model in SPM8 to facilitate optimal segmentation^51^.

Next, a group-level analysis was conducted to evaluate the relationship between an individual’s total score on the PTGI or the subscore for relating to others and the delta-rGMV or rGMV at the post-assessment at the whole-brain level using voxel-by-voxel multiple regression analyses performed with SPM8. It is standard practice to control for individual differences in overall head size by including the total intracranial volume (TIV) as a covariate^52^ because global normalization is about dealing with brains of different sizes in different populations^53^. Furthermore, we treated sex, age, total scores on the RAPM, the total scores of the CAPS, CES-D, the T-A subscale of the STAI, and the interval between the pre- and post-MRI data acquisitions as additional confounding variables to rule out all potential confounding factors that may affect the VBM findings.

We can aim to test a specific hypothesis about the relationships with certain regions of interests (ROIs) by enclosing the expected volume change associated with PTG, based on previous volumetric studies related to PTG. In those cases, it is appropriate to apply a small volume correction (SVC) that is restricted to the multiple tests performed within the ROIs. Accordingly, a SVC was applied to the abovementioned multiple regression analyses using a uniform anatomical mask for brain regions that are functionally and structurally related to the PFC, based on the *a priori* hypothesis of the present study as follows: the Frontal_Sup_L/R, Frontal_Sup_Orb_L/R, Frontal_Mid_L/R, Frontal_Inf_Oper_L/R, Frontal_Inf_Tri_L/R, Cingulum_Ant_L/R, Cingulum_Mid_L/R, Cingulum_Post_L/R, Occipital_Mid_L/R, Parietal_Inf_L/R, Precuneus_L/R, Temporal_Sup_L/R, and Temporal_Mid_L/R. These regions were determined using the Anatomical Automatic Labeling (AAL) atlas^54^.

A TFCE with family-wise error (FWE) corrected to *P* < 0.05 was used to define the cluster and control for multiple comparisons over 5,000 permutations (default setting), based on a finding that 1,000 permutations resulted in a minimum possible *P*-value of 0.001^55^.

A regression analysis was conducted with the value of the peak voxel in the significant cluster of delta-rGMV as the dependent variable and sex; age; total scores on the RAPM, CAPS, and CES-D; score on the T-A subscale of the STAI; TIV; and the interval between the pre- and post-assessment data acquisitions as independent variables to determine whether the factors and effect sizes for the relationships between the delta-rGMVs were significantly associated with total score on the PTGI (or subscore for relating to others) and related factors. A significance level of 0.05 with a two-sided probability was applied to the analyses. Additionally, the multicollinearity among the variables in the multiple regression analyses was evaluated because this factor can have significant effects on the estimates of the parameters when calculating the variance inflation factor (VIF). For more details, see the Supplementary Methods section.

## Additional Information

**How to cite this article**: Nakagawa, S. *et al*. Effects of post-traumatic growth on the dorsolateral prefrontal cortex after a disaster. *Sci. Rep.* **6**, 34364; doi: 10.1038/srep34364 (2016).

## Supplementary Material

Supplementary Information

## Figures and Tables

**Table 1:** Pearson’s correlations among the PTGI factors, CAPS, CES-D, and Trait-Anxiety scale of the STAI.

	Mean (SD)	1	2	3	4	5	6	7
1. Relating to others	13.96 (8.43)	—						
2. New possibilities	8.46 (5.45)	0.837*	—					
3. Personal strength	7.73 (4.55)	0.624*	0.715*	—				
4. Spiritual change and appreciation of life	5.85 (4.13)	0.726*	0.733*	0.691*	—			
5. CAPS	7.73 (11.09)	0.144	−0.023	−0.219	0.113	—		
6. CES-D	11.65 (10.42)	−0.076	−0.156	−0.449	−0.261	0.321	—	
7. Trait-Anxiety	42.58 (9.77)	−0.002	−0.122	−0.469	−0.126	0.388	0.820*	—

*P < 0.01 (two-tailed) after Bonferroni’s correction. Abbreviations: CAPS, Clinician-administered PTSD Scale; CES-D, Center for Epidemiologic Studies Depression Scale; PTGI, Post-traumatic Growth Inventory; STAI, State–Trait Anxiety Inventory.

**Table 2:** Determinants of the delta-rGMV value of the peak voxel in the significant cluster: multiple regression analyses.

Dependent variables	Independent variables	*R*	Adjusted *R*^2^	*F*	*β*	VIF
The delta-rGMV value of the peak voxel (54, 38, 25) in the significant cluster	Relating to others	0.890	0.674	6.738**	0.778***	1.271
	CAPS				−0.428*	1.711
	CES-D				0.173	3.451
	Trait-anxiety				−0.123	3.615
	Sex				0.103	3.152
	Age				0.249	1.613
	RAPM				0.721***	1.374
	Total intracranial volume				0.093	3.840
	Interval between the pre- and post-assessments				−0.336*	1.558
The delta-rGMV value of the peak voxel (54, 42, 22) in the significant cluster	PTG	0.852	0.573	4.724	0.744**	1.219
	CAPS				−0.418*	1.696
	CES-D				0.309	3.449
	Trait-anxiety				−0.196	3.585
	Sex				0.073	3.069
	Age				−0.013	1.610
	RAPM				0.680***	1.354
	Total intracranial volume				−0.162	3.805
	Interval between the pre- and post-assessments				−0.269	1.554

Significance: *P < 0.05, **P < 0.01, ***P < 0.001. Abbreviation: CAPS, Clinician-administered PTSD Scale; CES-D, Center for Epidemiologic Studies Depression Scale; delta-rGMV, delta- regional grey matter volume; DLPFC, dorsolateral prefrontal cortex; PTG, post-traumatic growth; RAPM, Raven’s Advanced Progressive Matrix; VIF, variance inflation factor.
