# Supplementary material for: Effects of post-traumatic growth on the dorsolateral prefrontal cortex after a disaster
Source: Sci Rep. 2016 Sep 27;6:34364. doi: 10.1038/srep34364 (PMC5037468; doi:10.1038/srep34364)
Supplement: Supplementary Information [file srep34364-s1.doc]

**Effects of post-traumatic growth on the dorsolateral prefrontal cortex after a disaster**

**Seishu Nakagawa ^1,2,*^, Motoaki Sugiura ^1,3^, Atsushi Sekiguchi ^1,4,5^, Yuka Kotozaki ^1^, Carlos Makoto Miyauchi ^1,6^, Sugiko Hanawa^1^, Tsuyoshi Araki ^7^, Hikaru Takeuchi ^1^, Atsushi Sakuma ^1,8^, Yasuyuki Taki ^1,4^ , Ryuta Kawashima ^1^**

^1^ Institute of Development, Aging and Cancer (IDAC), Tohoku University, Sendai, Japan

^2^ Department of Psychiatry, Tohoku Medical and Pharmaceutical University, Sendai, Japan

^3^ International Research Institute of Disaster Science, Tohoku University, Sendai, Japan

^4^ Division of Medical Neuroimage Analysis, Department of Community Medical Supports,

Tohoku Medical Megabank Organization, Tohoku University, Sendai, Japan

^5^ Department of Adult Mental Health, National Institute of Mental Health, National

Center of Neurology and Psychiatry, Kodaira, Tokyo, Japan.

^6^ Graduate School of Arts and Sciences, The University of Tokyo, Tokyo, Japan.

^7^ Advantage Risk Management Co., Ltd., Tokyo, Japan

^8^ Department of Psychiatry, Tohoku University Graduate School of Medicine, Sendai, Japan

**Supplementary Methods**

**Image acquisition**

The high grey-white matter contrast and spatial resolution provided by T1-weighted MRI is a standard, widely used imaging protocol for computational studies of brain anatomy ^1^. Magnetization Prepared Rapid Acquisition Gradient Echo (MPRAGE)-based T1 mapping is rapid, accurate and precise ^2^.

**Analysis**

***Neuroimaging data analysis***

Voxel-based morphometry (VBM) is a commonly used tool for detecting regional changes in the brain in response to neurological and psychiatric dysfunction, and can even detect their characteristics in healthy subjects ^3^. As described by Ashburner et al, the acquired raw brain images must be spatially normalized, segmented into different tissue classes, and smoothed before the statistical tests. The pre-processing step involves spatially normalizing the subjects’ brain images to a standard template in stereotactic space by matching the grey matter (GM) in these images to a grey matter reference, based on the prior probability maps ^4^. The morphological data were preprocessed with Statistical Parametric Mapping (SPM) using the default parameter settings, except that affine regularization was performed with the International Consortium for Brain Mapping template for East Asian brains. Then, GM, white matter (WM), and cerebrospinal fluid (CSF) population templates were generated from the entire image dataset with diffeomorphic anatomical registration using the exponential Lie algebra (DARTEL) technique incorporated in SPM8. Nonlinear registration is considered a local optimization problem, which is solved using the Levenberg-Marquardt strategy. Finally, the images were smoothed with an 8-mm full width at half-maximum isotropic Gaussian kernel. After spatial preprocessing, the smoothed, modulated, normalized GM and WM datasets were subjected to statistical analysis. Then, the change in the rGMV signals between the pre- and post-images were computed for each voxel in each participant. This computation only included the voxels that had GMV values >0.10 in both the pre- and post-MRI scans to avoid possible partial volume effects around the borders between the grey matter and white matter and between the grey matter and CSF. The resulting maps representing delta-rGMV (the rGMV change from pre- to post-assessment) were then forwarded to the group-level analysis described below. See Takeuchi et al for additional details about the preprocessing procedure using SPM8 ^5^.

The WFU_PickAtlas software toolboxes ^6, 7^ provide a method for generating region-of-interest (ROI) masks based on the Talairach Daemon database. The Anatomical Automatic Labeling (AAL) atlas was included in the PickAtlas toolbox as a subset of structural ROIs. The AAL atlas was spatially normalized to the single-subject high-resolution T1 volume ^8^ and was used by researchers of the neuroimaging community. The atlases not only include Brodmann areas but also information from other atlases, including the AAL atlas ^8^, which have been used in previous VBM studies ^9, 10^.

Cluster-based thresholding is popular, as it is more sensitive to finding the true signal than voxel-wise thresholding, whereas cluster-based inference must account for nonstationarity in VBM. Threshold-free cluster enhancement (TFCE) takes a raw, static image and produces an output image in which the voxel-wise values represent the amount of cluster-like local spatial contiguity. The TFCE image can be transformed into voxel-wise *P*-values via permutation testing against the false positive rate ^11^. Thus, the TFCE inference is fairly robust in response to non-stationarity in the data and improved the sensitivity and interpretable output ^11^.

**Supplementary References**

1. Lorio, S. et al. Neurobiological origin of spurious brain morphological changes: A quantitative MRI study. *Human brain mapping* **37**, 1801-1815 (2016).

2. Liu, J.V., Bock, N.A. & Silva, A.C. Rapid high-resolution three-dimensional mapping of T1 and age-dependent variations in the non-human primate brain using magnetization-prepared rapid gradient-echo (MPRAGE) sequence. *NeuroImage* **56**, 1154-1163 (2011).

3. Ridgway, G.R. et al. Ten simple rules for reporting voxel-based morphometry studies. *NeuroImage* **40**, 1429-1435 (2008).

4. Good, C.D. et al. A voxel-based morphometric study of ageing in 465 normal adult human brains. *NeuroImage* **14**, 21-36 (2001).

5. Takeuchi, H. et al. The Associations between Regional Gray Matter Structural Changes and Changes of Cognitive Performance in Control Groups of Intervention Studies. *Front Hum Neurosci* **9**, 681 (2015).

6. Maldjian, J.A., Laurienti, P.J., Kraft, R.A. & Burdette, J.H. An automated method for neuroanatomic and cytoarchitectonic atlas-based interrogation of fMRI data sets. *NeuroImage* **19**, 1233-1239 (2003).

7. Maldjian, J.A., Laurienti, P.J. & Burdette, J.H. Precentral gyrus discrepancy in electronic versions of the Talairach atlas. *NeuroImage* **21**, 450-455 (2004).

8. Tzourio-Mazoyer, N. et al. Automated anatomical labeling of activations in SPM using a macroscopic anatomical parcellation of the MNI MRI single-subject brain. *NeuroImage* **15**, 273-289 (2002).

9. Kasai, K. et al. Evidence for acquired pregenual anterior cingulate gray matter loss from a twin study of combat-related posttraumatic stress disorder. *Biol Psychiatry* **63**, 550-556 (2008).

10. van Wingen, G.A., Geuze, E., Vermetten, E. & Fernandez, G. Perceived threat predicts the neural sequelae of combat stress. *Mol Psychiatry* **16**, 664-671 (2011).

11. Smith, S.M. & Nichols, T.E. Threshold-free cluster enhancement: addressing problems of smoothing, threshold dependence and localisation in cluster inference. *NeuroImage* **44**, 83-98 (2009).
